# Inferring Pathway Activity toward Precise Disease Classification

**DOI:** 10.1371/journal.pcbi.1000217

**Published:** 2008-11-07

**Authors:** Eunjung Lee, Han-Yu Chuang, Jong-Won Kim, Trey Ideker, Doheon Lee

**Affiliations:** 1 Department of Bio and Brain Engineering, KAIST, Daejeon, South Korea; 2 Bioinformatics Program, University of California San Diego, La Jolla, California, United States of America; 3 Department of Bioengineering, University of California San Diego, La Jolla, California, United States of America; 4 Department of Laboratory Medicine and Genetics, Sungkyunkwan University, School of Medicine, Samsung Medical Center, Seoul, South Korea; Lilly Singapore Centre for Drug Discovery, Singapore

## Abstract

The advent of microarray technology has made it possible to classify disease states based on gene expression profiles of patients. Typically, marker genes are selected by measuring the power of their expression profiles to discriminate among patients of different disease states. However, expression-based classification can be challenging in complex diseases due to factors such as cellular heterogeneity within a tissue sample and genetic heterogeneity across patients. A promising technique for coping with these challenges is to incorporate pathway information into the disease classification procedure in order to classify disease based on the activity of entire signaling pathways or protein complexes rather than on the expression levels of individual genes or proteins. We propose a new classification method based on pathway activities inferred for each patient. For each pathway, an activity level is summarized from the gene expression levels of its condition-responsive genes (CORGs), defined as the subset of genes in the pathway whose combined expression delivers optimal discriminative power for the disease phenotype. We show that classifiers using pathway activity achieve better performance than classifiers based on individual gene expression, for both simple and complex case-control studies including differentiation of perturbed from non-perturbed cells and subtyping of several different kinds of cancer. Moreover, the new method outperforms several previous approaches that use a static (i.e., non-conditional) definition of pathways. Within a pathway, the identified CORGs may facilitate the development of better diagnostic markers and the discovery of core alterations in human disease.

## Author Summary

The advent of microarray technology has drawn immense interest to identify gene expression levels that can serve as biomarkers for disease. Marker genes are selected by examining each individual gene to see how well its expression level discriminates different disease types. In complex diseases such as cancer, good marker genes can be hard to find due to cellular heterogeneity within the tissue and genetic heterogeneity across patients. A promising technique for addressing these challenges is to incorporate biological pathway information into the marker identification procedure, permitting disease classification based on the activity of entire pathways rather than simply on the expression levels of individual genes. However, previous pathway-based methods have not significantly outperformed gene-based methods. Here, we propose a new pathway-based classification procedure in which markers are encoded not as individual genes, nor as the set of genes making up a known pathway, but as subsets of “condition-responsive genes (CORGs)” within those pathways. Using expression profiles from seven different microarray studies, we show that the accuracy of this method is significantly better than both the conventional gene- and pathway- based diagnostics. Furthermore, the identified CORGs may facilitate the development of effective diagnostic markers and the discovery of molecular mechanisms underlying disease.

## Introduction

Analysis of genome-wide expression profiles has become a widespread technique for identifying diagnostic markers of various disease states, outcomes, or responses to treatment [1]–[5]. Markers are selected by scoring each individual gene for how well its expression pattern can discriminate between different classes of disease or between cases and controls. The disease status of new patients is predicted using classifiers tuned to the expression levels of the marker genes.

One challenge of expression-based classification is that cellular heterogeneity within tissues and genetic heterogeneity across patients in complex diseases may weaken the discriminative power of individual genes [6]–[9]. In addition, marker genes are typically selected independently although proteins are known to function coordinately within protein complexes, signaling cascades, and higher-order cellular processes. Thus, the resulting expression-based classifiers may contain unnecessarily many marker genes with redundant information which may lead to decreased classification performance [10].

Due to these types of difficulties, several groups have hypothesized that a more effective means of marker identification may be to combine gene expression measurements over groups of genes that fall within common pathways [11]–[17]. The pre-defined functional groupings of genes are drawn from canonical pathways curated from literature resources such as the Gene Ontology [18] and KEGG databases [19] or experimentally defined gene lists from microarray studies [15],[16],[20]. Recently, pathway-based analysis has been extended to perform disease classification of expression profiles. Some approaches use gene expression parametrically by representing pathway activity with a function summarizing the expression values of member genes [21],[22], while others estimate probabilities of pathway activation based on the consistency of changes in gene expression [23],[24]. Alternative approaches engineer normal cells to activate pre-selected oncogenic pathways to determine gene signatures which can distinguish tumor characteristics [20],[25]. These methods have demonstrated classification accuracies that are comparable to conventional gene-based classifiers, while providing a strong biological interpretation for why the expression profile is associated with a particular type of disease (i.e., based on the pathways found to be perturbed). On the other hand, a potential shortcoming of current pathway-based classifiers is that the pre-defined set of genes making up a pathway may be derived from conditions irrelevant to the disease of interest. Moreover, not all the member genes in a perturbed pathway are typically altered at the mRNA level.

Here, we propose a novel gene-expression-based diagnostic that incorporates pathway information in a condition-specific manner (Pathway Activity inference using Condition-responsive genes, PAC). The markers are encoded not as individual genes, nor as static literature-curated pathways, but as subsets of condition-responsive co-functional genes (Condition-Responsive Genes, CORGs). To optimally discriminate samples of different phenotypes, we identify CORGs from each static pathway in the context of the specific disease in question. The combined expression levels of the CORGs are treated as the pathway “activity” and used to build classifiers for predicting the disease status of new patients. We show that our pathway-based approach outperforms previous analyses of differential expression in classifying samples across seven different datasets. Moreover, we show that pathway activities inferred using only CORGs lead to better classification performance as compared to pathway activities inferred using various types of summary statistics of all genes which participate in a common pathway. The resulting pathway markers and their CORGs also provide models of the molecular mechanisms which define the disease of interest.

## Methods

### Datasets

We obtained previously published mRNA expression datasets covering seven different disease classification scenarios: 24 expression profiles of HeLa cells after stimulation by Tumor Necrosis Factor (TNF) [26], expression profiles of 62 primary prostate tumors and 41 normal prostate specimen [27], expression profiles of 143 acute lymphoblastic leukemia (ALL) patients [28], breast cancer expression profiles for 295 patients from the Netherlands [29] and 286 patients from the USA [5], and lung cancer expression profiles for 86 patients from Michigan [30] and 62 patients from Boston [31].

Each dataset was divided into two populations of distinct phenotypes as per the original publications (Table S1). For the TNF study [26], 12 samples had normal IkB proteins (labeled “Wildtype”) and 12 samples expressed mutant IkB blocking NF-kB signaling (labeled “Mutant”). For the prostate cancer study [27], 62 samples were retrieved from primary tumors (labeled “Cancer”) and 41 samples were from normal prostate specimen (labeled “Normal”). For the ALL study [28], 79 patients suffered from one subtype resulting from a t(12;21)(p12,q22) reciprocal translocation (labeled “TEL-AML1”) and the other 64 patients showed hyperdiploid hyperdip >50 (labeled “HH”). For the two breast cancer datasets, metastasis had been detected in 78 [29] and 106 [5] patients during follow-up visits within five and seven years after surgery (labeled “Metastatic”); the remaining 217 and 180 patients were still metastasis free (labeled “Non-metastatic”). For the two lung cancer datasets, we defined the two phenotype populations according to Subramanian et al. [15], who labeled 24 patients in the Michigan dataset and 31 patients in the Boston dataset as having a “Poor” prognosis, while the remaining 62 and 31 patients were labeled as having a “Good” prognosis.

For pathway information, we used the C2 functional set downloaded from MsigDB v1.0 [15]. This set includes 472 canonical metabolic and signaling pathways pooled from eight manually curated databases along with 50 co-expressed gene clusters obtained from various microarray studies. Each pathway or gene cluster defines a set of genes (gene clusters are henceforth also called “pathways”). In total, the available pathways covered 5602 genes, most but not all of which were measured in the seven gene expression datasets, due to the various array platforms used.

### Condition-Responsive Gene Identification and Pathway Activity Inference

**Figure 1:**
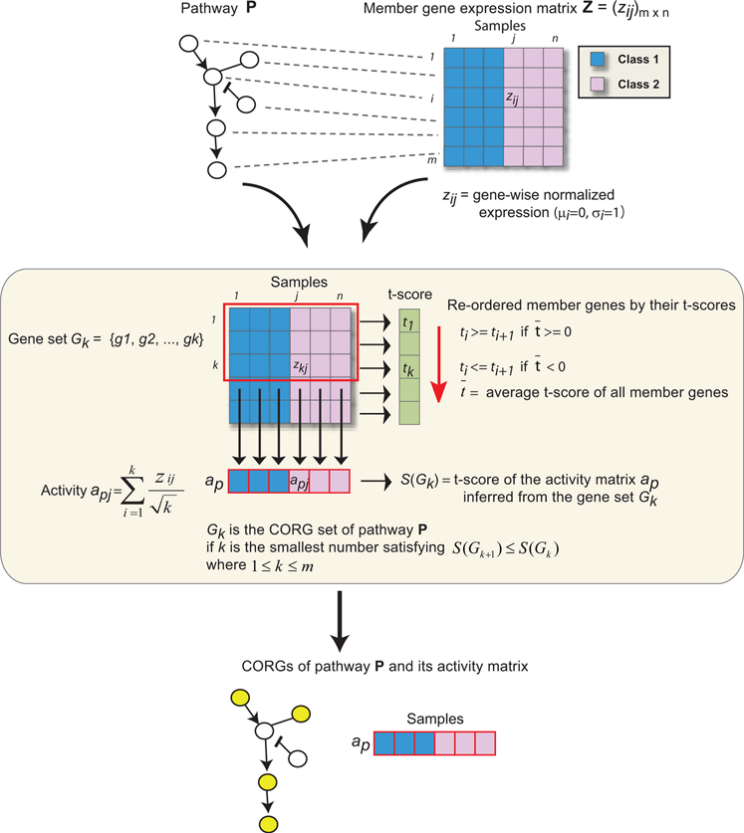
A schematic diagram of key gene identification and activity inference. Selected significant pathways are further subject to CORG identification corresponding to the phenotype of interest. Gene expression profiles of patient samples drawn from each subtype of diseases (e.g., good or poor prognosis) are transformed into a “pathway activity matrix”. For a given pathway, the activity is a combined z-score derived from the expression of its individual key genes. After overlaying the expression vector of each gene on its corresponding protein in the pathway, key genes which yield most discriminative activities are found via a greedy search based on their individual power (see Methods). The pathway activity matrix is then used to train a classifier.

To integrate the expression and pathway datasets, we overlaid the expression values of each gene on its corresponding protein in each pathway. Within each pathway, we searched for a subset of member genes whose combined expression levels across the samples were highly discriminative of the phenotypes of interest (Figure 1). For a particular gene set ***G***, let ***a*** represent its vector of activity scores over the samples in a study, and let ***c*** represent the corresponding vector of class labels (e.g. good vs. poor prognosis). To derive ***a***, expression values ***g_ij_*** are normalized to z-transformed scores ***z_ij_*** which for each gene ***i*** have mean μ_i_ = 0 and standard deviation σ_i_ = 1 over all samples ***j***. The individual ***z_ij_*** of each member gene in the gene set are averaged into a combined *z*-score which is designated the activity ***a_j_*** (the square root of the number of member genes is used in the denominator to stabilize the variance of the mean). Many types of statistic, such as the Wilcoxon score or Pearson correlation, could be used to score the relationship between ***a*** and ***c***. In this study, we defined the discriminative score ***S***(***G***) as the *t*-test statistic [32] derived on ***a*** between groups of samples defined by ***c***.

For a given pathway, a greedy search was performed to identify a subset of member genes in the pathway for which ***S(G)*** was locally maximal. We refer to this subset as the set of “condition-responsive genes” (CORGs) representing the majority of the pathway activation under the relevant conditions. To identify the CORG set, member genes were first ranked by their *t*-test scores, in ascending order if the average *t*-score among all member genes was negative, and in descending order otherwise. The CORG set ***G*** was initialized to contain only the top member gene and iteratively expanded. At each iteration, addition of the gene with the next best *t*-test score was considered, and the search was terminated when no addition increased the discriminative score ***S*** **(** ***G*** **)**. The activity vector ***a*** of the final CORG set was regarded as the pathway activity across the samples.

### Previous Gene-Set Ranking Approaches and Other Pathway-Based Classification Methods

We also used a method proposed by Tian et al. [16] to assess the probability of a pathway being altered in disease based on the correlation between the expression of all its member genes and the disease phenotype. For each pathway *P* in MsigDB, Tian et al. calculated a score *T* by averaging the *t*-test statistic scores of all member genes. Higher *T* was indicative of stronger pathway correlation with the disease status. The top 10% of pathways (52 pathways) in each dataset were selected for further analysis and for classification. The decision of whether a pathway had been disrupted by disease was assessed on the basis of the discriminating power of the member genes between the classes of interest (using a *t*-test statistic). However, there may be some signatures of pathway disruption that are independent of the classification task at hand. To detect such signatures, a number of statistical functions [8],[33] can be adopted in the framework of Tian et al. Unlike the *t*-test, these functions are designed to detect perturbed patterns rather than mean expression changes.

To compare our PAC with other activity inference schemes, we implemented three other expression summarization methods, including a principal component analysis (PCA) similar to that used in Bild et al. [20] and the mean and median approaches used in Guo et al. [22]. Bild et al. used the first principal component of the expression of the member genes to represent the activation of a given pathway, while Guo et al. summarized the expression levels of member genes by using simple statistics like mean and median.

### Marker Robustness Evaluation

For each dataset, 100 alternative two-fold splits were generated of each mRNA expression profile in the dataset. Pathways were ranked on each fold using the method of Tian et al. [16], and CORGs for each pathway were identified using the samples in a single fold. Individual genes were also ranked by their discriminative power on each fold. The robustness was estimated as the average degree of overlap among top pathways/genes derived from the two folds of samples across the 100 splits.

### Classification Evaluation

Logistic regression models [34] were trained on both the pathway activity matrix (pathways versus samples) and the original gene expression matrix (genes versus samples—i.e., conventional gene-based classification). For within-dataset experiments, the expression samples in a dataset were divided so that four-fifths of the samples were used as the training set to build the classifier, and one fifth were used as the test set (five-fold cross validation). Each of the five subsets in the dataset was evaluated in turn as the test set and withheld during marker selection (including CORG identification) and classifier training. In order to train a generalized classifier and to minimize over-fitting, we further split the training set into three smaller subsets of equal size: two subsets were used as the marker selection set to rank markers (pathways or genes) as well as identify CORGs (pathways only), and one subset was used as the validation set for assessing which marker set was significant for classification. Thus the CORGs might be different for a specific pathway, depending on the samples used in the marker selection set. Pathways or genes were ranked by the *p*-value of discriminative power to classify samples in the marker selection set, after which the logistic regression model was built by adding markers sequentially in increasing order of *p*-value (sequential selection). The number of markers used in the classifier was optimized by evaluating its Area Under ROC Curve (AUC, see [35] for details) on the validation set. The AUC metric captured performance over the entire range of sensitivity/specificity values. The final classification performance was reported as the AUC on the test set using the classifier optimized from the validation set. For unbiased evaluation, we generated 100 alternative five-fold splits of samples in each dataset and ran cross validation on each split. The final reported AUC values were averaged across 500 randomly selected ways of partitioning the data into four-fifths training and one-fifth test samples.

For cross-dataset experiments, markers (pathways or genes) were selected using the whole first dataset and then tested on the second dataset (or vice versa). CORG identification was also performed on the first dataset. As for the within-dataset experiments, the patient samples in the second dataset were divided into five subsets of equal size: four subsets were designated as the “training” set to build the classifier using markers from the first dataset, and one subset was held for testing. One hundred alternative five-fold splits were generated to partition samples in the second dataset into four-fifths for training and one-fifth for testing. Therefore, we learned 500 classifiers for each of these two datasets, in which each classifier was associated with its own pathway marker set. The averaged AUC values among the 500 classifiers built on the second dataset were reported as the final classification performance for each marker set identified from the first dataset. Among the 500 classifiers, the pathway marker set used in classification could be different depending on which training samples were used in the second dataset. However, the CORGs of each pathway were the same across these 500 classifiers because the identification was done using the whole first dataset.

In this study, for pathway-based classifiers, the input marker set was defined as the top 10% of pathways in MSigDB ranked by Tian et al. [16] using a designated training set. In order to compare pathway and gene based methods in a fair manner that controls for the number of genes used, we provided the gene-based classifiers with the same number of top ranked genes as the number of CORGs pooled from the significant pathways selected by Tian et al. [16].

## Results/Discussion

### Pathway Markers Amplify Signals over Multiple Weak Gene Markers

We first tested the robustness of the pathway markers selected by the method of Tian et al. [16]. The agreement between the significant pathways was higher than that between the individually scored gene markers (Figure S1). The CORGs within the top pathways were also more consistent than individually scored gene markers in different subsets of samples. The observed robustness of CORGs might imply that some non-differentially expressed genes, which are often dropped in conventional analysis, do have associations with the disease of interest.

**Figure 2:**
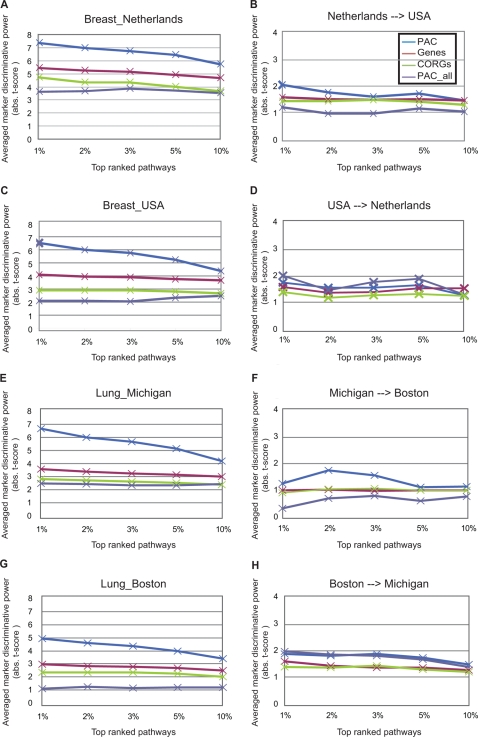
Discriminative power of pathway and gene markers in the breast and lung cancer datasets. Mean absolute *t*-scores against phenotypes were compared between four marker sets in the source dataset, which was used to identify markers—(A) and (C) for the two breast cancer datasets and (E) and (G) for the two lung cancer datasets—or in an independent verification dataset—(B) (D) (F) (H). Pathway markers were ranked by using their absolute *t*-scores from a two-tail *t*-test on activity levels (see *S(G)* in Methods) between the two phenotypes of interest in the source dataset, and their discriminative power in the same order was measured in the verification dataset. Pathway activities were estimated using only CORGs (PAC) or all member genes (PAC_all). The individual predictive power of CORGs in the top pathways was also evaluated using the same *t*-test on their gene expression levels (CORGs). A similar analysis was performed using the same number of top discriminative genes as the number of CORGs covered by the pathway markers (Genes).

We hypothesized that pathway information could be used to restrict the search space for truly perturbed genes whose aggregated expression is more predictive for disease status than individually considered. We began by analyzing the breast and lung cancer datasets (four datasets in total), since each dataset has available two separate cohorts of patients studied by different researchers. The top 10% of pathways were selected for each of the four datasets (see Methods). We identified the CORGs for each top pathway and aggregated their expression levels into a single activity value for each sample (Methods). By design, the inferred pathway activities had more discriminative power in distinguishing samples with different disease phenotypes than did the individual expression levels of the member CORGs (PAC versus CORGs in Figure 2A, 2C, 2E, and 2G). However, the discriminative power fell when the pathway activity was inferred using not only the CORGs but all member genes associated with each pathway (PAC_all in Figure 2A, 2C, 2E, and 2G). This result suggests that, as might be expected, not all genes in a significant pathway are transcriptionally altered or associated with the phenotype of interest.

We then compared our pathway markers to the individual gene markers selected without pathway information. We found that the PAC activity scores outperformed individual gene markers in terms of discriminating samples with different disease phenotypes in both the source datasets used for marker identification (PAC versus Genes in Figure 2A, 2C, 2E, and 2G) and the independent verification datasets (Figure 2B, 2D, 2F, and 2H). In the verification datasets, the CORGs demonstrated almost the same discriminative power as did the top genes, although the top genes were more powerful in the original datasets. These comparisons suggest that aggregating the perturbed genes in a pathway leads to a better marker for discriminating disease phenotypes. Although the expression of a single gene might not be a strong predictor, pathway integration provides a means to amplify individual weak signals at the transcriptional level.

### Pathway Markers Increase the Classification Accuracy

We next tested that the inferred pathway activity levels could be used in the classification of disease status for a new expression profile. To use pathway information for classification, pathway activities were used as feature values in a classifier based on logistic regression. The technique of five-fold cross validation was applied to test the predictive power of the pathway markers (see Methods). In each run of cross validation, we only considered the top 10% of pathway markers selected by Tian et al. [16] using the designated training data.

**Figure 3:**
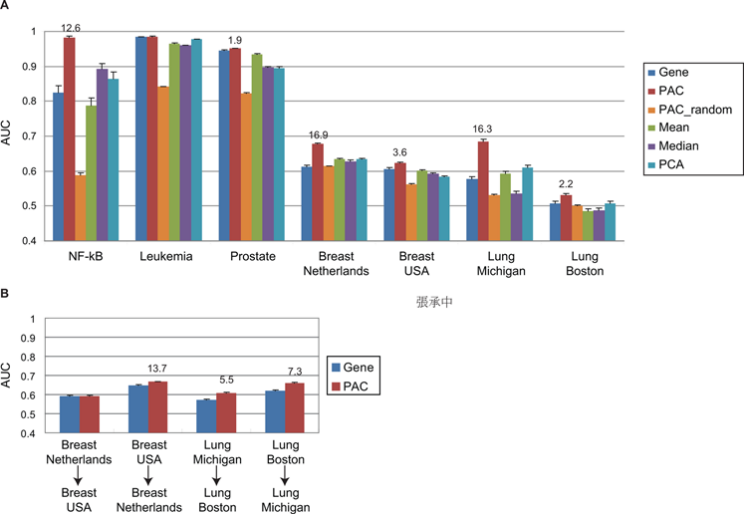
Classification accuracy within (A) and across (B) datasets. Bar chart of Area Under ROC Curve (AUC) classification performance of CORG-based pathway markers (PAC), conventional pathway markers (Mean, Median, and PCA), and individual genes (Gene; same number of top discriminative genes as the number of CORGs in pathway markers). Classification performance is summarized as mean±ste of AUC over 100 runs of 5-fold cross-validation within a dataset. To compute PAC_random, the AUC values of 1000 sets of random gene sets were averaged. Numbers above the red bars are -log (*p*-value) from the Wilcoxon signed-rank test on the 500 AUCs of “PAC” against those of “Gene” (only the ones with *p*-value<0.05 are shown). The *p*-values measure the significance of difference between PAC and gene-based classification.

As shown in Figure 3A, our pathway-based classifiers (PAC) significantly outperformed the conventional gene-based classifiers (Gene). The improved performance was not simply due to grouping multiple gene expression measurements, as shown by comparing our performance with that of random groups of genes (PAC_random; averaged AUCs of 1000 sets of same-size random gene sets as the significant pathways). Classifiers using pathway activity inferred by the mean or median of the member gene expression [22] or the 1^st^ principle component (PCA) [20] had higher predictive power than those using random gene sets (PAC_random), but only comparable power to the conventional gene-based classifiers. These results indicate that there are at least two critical factors in developing an advanced molecular diagnostic: (1) a biologically meaningful definition of pathways and (2) inference of condition-specific pathway activity.

Next, we tested the reproducibility of the pathway markers selected across different microarray platforms or different cohorts of patients. For this purpose, we used expression profiles of the two lung cancer datasets and the two breast cancer datasets generated from different groups. For each cancer, significant pathways and their CORGs were identified using the whole first dataset and then tested on the second dataset, or vice versa (Figure 3B). Our pathway-based classifiers again significantly outperformed the gene-based classifiers.

To show that the better performance of PAC was not dependent on the chosen classification algorithm, we evaluated all markers and pathway activity inference methods using three additional classification approaches: k-nearest neighbors, naïve Bayes, and linear discriminative analysis. Moreover, forward selection method was also employed to show our superior performance was not beneficial from the feature selection method used. All further analyses demonstrated the same trends, i.e., our CORG-based pathway classifiers outperformed other gene-based and pathway-based classifiers (Figures S2 and S3).

### Pathway Markers and Their CORGs Provide Biologically Informative Models for Lung Cancer Prognosis

Beyond achieving better classification performance, the discriminative pathway markers and their CORGs can lend insight into the biological basis for why samples are classified as a specific disease status. As an example, we examined the pathway markers selected in the above two cross-dataset experiments for classification of lung cancer prognosis (for a similar analysis of breast cancer metastasis, see Table S2 and Figure S4). We counted the frequency with which each pathway in MSigDB was selected over the 500 classifiers, and we identified the top most frequent pathways having over 100 occurrences (Table 1).

**Table 1:** Frequently selected pathway markers for lung cancer prognosis.

Pathway Name	Frequency	# genes^a^	CORGs
**From Michigan to Boston**			
Glutamine up-regulated genes	433/500	5/313	NP LDHA BZW1 TUBA1 LAMB3
Gluconeogenesis	247/500	2/32	LDHA ENO2
*Glycolysis* b	245/500	3/22	*ENO2 PGK1* ALDOA
*Breast cancer estrogen signaling*	203/500	3/101	VEGF *KRT18 KRT19*
Glycolysis and gluconeogenesis	176/500	5/55	GAPD LDHA ENO2 ALDH3B2 ALDH3B1
*Estrogen receptor modulators down-regulated genes*	138/500	4/74	ARHE STC1 *KRT7* COPEB
Leucine down-regulated genes	134.500	4/180	NP LDHA TUBA1 CCNA2
B lymphocyte pathway	102/500	4/11	CR2 ITGAL HLA-DRA CR1
**From Boston to Michigan**			
*Breast cancer estrogen signaling*	481/500	6/101	*KRT18 KRT19* GAPD MT3 CDKN2A TFF1
Pyrimidine metabolism	258/500	3/45	POLR2E NP RRM1
*Glycolysis*	258/500	2/22	*ENO2 PGK1*
MTA3 pathway	238/500	3/16	TUBA1 GAPD MTA1
Insulin up-regulated genes	165/500	10/235	PGAM1 ARF4 ARCN1 DNCL1 EIF2S2 PSMA6 YWHAH PSMA3 ZNF9 CLNS1A
P53 hypoxia pathway	148/500	3/20	FHL2 IGFBP3 HIF1A
Glutamine down-regulated genes	133/500	4/313	PGAM1 ERH PAICS BZW1
p53 signalling	114/500	6/101	HIF1A FADD GAPD APEX1 CDKN2A CSNK2B
*Estrogen receptor modulators down-regulated genes*	108/500	3/74	*KRT7* DUSP4 MMD
NFKB up-regulated genes	103/500	2/111	KRT7 GBP1

a The number of CORGs and member genes are specified.

b Pathways/Genes in italics are shared between datasets.

**Figure 4:**
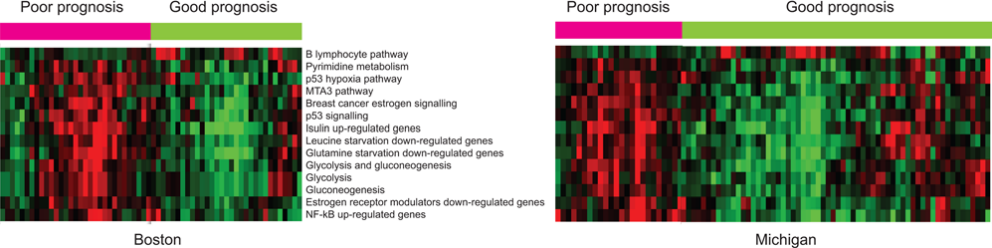
Pathway activity of the top frequently used markers in the two lung cancer datasets. Activities were inferred from CORGs identified from each dataset. Green/red blocks indicate pathways (rows) that are up-/down- regulated in patients (columns) of specific prognosis (above color bars: pink and green indicate poor and good prognosis, respectively). Pathways are clustered based on the similarity of their activities across patients.

Pathways involved in glucose metabolism (“Glycolysis” in Table 1) and estrogen signaling (“Breast cancer estrogen signaling” and “Estrogen receptor modulators down-regulated genes”) were frequently used in classifying lung cancer patients, and over-expression of these pathways had poor prognosis in both datasets (Figure 4). Constitutively up-regulated glycolysis has been observed in most primary and metastatic cancers and further explored to develop potential therapeutic targets [36]–[38]. Up-regulated glycolysis enables unconstrained proliferation and invasion and may lead to a more aggressive type of lung cancer [37]. Estrogen signaling has been known to promote cell proliferation and suppresses apoptosis, and its role in the late steps of lung metastasis has recently been suggested [39]. As shown in Table 1, many pathways could be represented by CORGs of the size from two to four, although some required more than eight genes (Figure S5). Especially for larger CORG sets, it would be computationally infeasible to identify these combinations to have maximal discriminative power in the absence of prior pathway knowledge.

### Conclusion

We have demonstrated that effectively incorporating pathway information into expression-based disease diagnosis can provide better discriminative and more biologically defensible models. Grouping gene expression responses via functional linkages can amplify individually weak signals due to the heterogeneity of samples, either genetic or technical. In addition, such gene groupings also emerge as a critical step of removing potential redundancy on expression among genes associated with the same function. In view of classification tasks, genes of the same expression pattern do not provide extra information for a classifier but may cause over-fitting. The identification of condition-responsive genes within each pathway helps to reduce noisy or variable measurements, leading to a more precise and robust classifier. Better coverage and quality of human pathway information is likely to enable more precise prediction of disease status and, accordingly, better management of patient care. In addition, human interaction databases are growing exponentially at present, enabling further opportunities for unveiling novel functional pathways or complexes [40]–[43]. Integrating known pathways and novel hypotheses from protein networks with expression profiles and phenotypic information will lead to more effective molecular characterization of human disease [17].

## Supporting Information

Table S1 The seven data sets used in method evaluation
(0.01 MB PDF)

Table S2 Frequently selected pathway markers for breast cancer prognosis
(0.01 MB PDF)

Figure S1 Marker reproducibility of pathway-based and gene-based selection in (A) NF-kB dataset, (B) Leukemia dataset, (C) Prostate dataset, (D) Netherlands dataset, (E) USA dataset, (F) Michigan dataset, and (G) Boston dataset Blue and yellow lines chart the magnitude of overlap among top n markers for pathways ranked by Tian et al. [16] and genes ranked by conventional *t*-test, respectively. Purple lines chart the magnitude of overlap among CORGs for the top n pathways. The performance of the 100 alternative splits is denoted by its mean.
(0.03 MB PDF)

Figure S2 Classification accuracy within and across datasets using different classifiers, (A) k-nearest neighbor with k = 3, (B) k-nearest neighbor with k = 5, (C) naïve Bayes and (D) linear discriminative analysis Bar charts denote classification accuracy in (A) and (B) and Area Under ROC Curve (AUC) in (C) and (D). Classification performance is summarized as mean +/− ste of accuracies/AUCs over 100 runs of 5-fold cross-validation. Numbers above the red bars are -log (*p*-value) from the Wilcoxon signed-rank test on the 500 accuracies/AUCs of “PAC” against those of “Gene” (only the ones with *p*-value<0.05 are shown).
(0.02 MB PDF)

Figure S3 Classification performance using sequential selection (SEQ) or forward selection (FWD)
(0.01 MB PDF)

Figure S4 Pathway activity of the top frequently used markers in the two breast cancer datasets Activities were inferred from CORGs identified from each dataset. Green/red blocks indicate pathways (rows) that are up-/down- regulated in patients (columns) of specific phenotype (above color bars: pink and green indicate metastasis and non-metastasis, respectively). Pathways are clustered based on the similarity of their activities across patients.
(0.11 MB PDF)

Figure S5 Distribution of numbers of CORGs in top 10% pathways
(0.01 MB PDF)

## References

[1] Alizadeh AA, Eisen MB, Davis RE, Ma C, Lossos IS (2000). Distinct types of diffuse large B-cell lymphoma identified by gene expression profiling.. Nature.

[2] Golub TR, Slonim DK, Tamayo P, Huard C, Gaasenbeek M (1999). Molecular classification of cancer: class discovery and class prediction by gene expression monitoring.. Science.

[3] Ramaswamy S, Ross KN, Lander ES, Golub TR (2003). A molecular signature of metastasis in primary solid tumors.. Nat Genet.

[4] van 't Veer LJ, Dai H, van de Vijver MJ, He YD, Hart AA (2002). Gene expression profiling predicts clinical outcome of breast cancer.. Nature.

[5] Wang Y, Klijn JG, Zhang Y, Sieuwerts AM, Look MP (2005). Gene-expression profiles to predict distant metastasis of lymph-node-negative primary breast cancer.. Lancet.

[6] Ein-Dor L, Kela I, Getz G, Givol D, Domany E (2005). Outcome signature genes in breast cancer: is there a unique set?. Bioinformatics.

[7] Symmans WF, Liu J, Knowles DM, Inghirami G (1995). Breast cancer heterogeneity: evaluation of clonality in primary and metastatic lesions.. Hum Pathol.

[8] Tomlins SA, Rhodes DR, Perner S, Dhanasekaran SM, Mehra R (2005). Recurrent fusion of TMPRSS2 and ETS transcription factor genes in prostate cancer.. Science.

[9] Mootha VK, Lindgren CM, Eriksson KF, Subramanian A, Sihag S (2003). PGC-1alpha-responsive genes involved in oxidative phosphorylation are coordinately downregulated in human diabetes.. Nat Genet.

[10] Saeys Y, Inza I, Larranaga P (2007). A review of feature selection techniques in bioinformatics.. Bioinformatics.

[11] Doniger SW, Salomonis N, Dahlquist KD, Vranizan K, Lawlor SC (2003). MAPPFinder: using Gene Ontology and GenMAPP to create a global gene-expression profile from microarray data.. Genome Biol.

[12] Draghici S, Khatri P, Martins RP, Ostermeier GC, Krawetz SA (2003). Global functional profiling of gene expression.. Genomics.

[13] Pavlidis P, Lewis DP, Noble WS (2002). Exploring gene expression data with class scores.. Pac Symp Biocomput.

[14] Pavlidis P, Qin J, Arango V, Mann JJ, Sibille E (2004). Using the gene ontology for microarray data mining: a comparison of methods and application to age effects in human prefrontal cortex.. Neurochem Res.

[15] Subramanian A, Tamayo P, Mootha VK, Mukherjee S, Ebert BL (2005). Gene set enrichment analysis: a knowledge-based approach for interpreting genome-wide expression profiles.. Proc Natl Acad Sci U S A.

[16] Tian L, Greenberg SA, Kong SW, Altschuler J, Kohane IS (2005). Discovering statistically significant pathways in expression profiling studies.. Proc Natl Acad Sci U S A.

[17] Chuang HY, Lee E, Liu YT, Lee D, Ideker T (2007). Network-based classification of breast cancer metastasis.. Mol Syst Biol.

[18] GO Gene Ontology Consortium

[19] Vert JP, Kanehisa M (2003). Extracting active pathways from gene expression data.. Bioinformatics.

[20] Bild AH, Yao G, Chang JT, Wang Q, Potti A (2006). Oncogenic pathway signatures in human cancers as a guide to targeted therapies.. Nature.

[21] Breslin T, Krogh M, Peterson C, Troein C (2005). Signal transduction pathway profiling of individual tumor samples.. BMC Bioinformatics.

[22] Guo Z, Zhang T, Li X, Wang Q, Xu J (2005). Towards precise classification of cancers based on robust gene functional expression profiles.. BMC Bioinformatics.

[23] Efroni S, Schaefer CF, Buetow KH (2007). Identification of key processes underlying cancer phenotypes using biologic pathway analysis.. PLoS ONE.

[24] Svensson JP, Stalpers LJ, Esveldt-van Lange RE, Franken NA, Haveman J (2006). Analysis of gene expression using gene sets discriminates cancer patients with and without late radiation toxicity.. PLoS Med.

[25] Glinsky GV, Berezovska O, Glinskii AB (2005). Microarray analysis identifies a death-from-cancer signature predicting therapy failure in patients with multiple types of cancer.. J Clin Invest.

[26] Tian B, Nowak DE, Jamaluddin M, Wang S, Brasier AR (2005). Identification of direct genomic targets downstream of the nuclear factor-kappaB transcription factor mediating tumor necrosis factor signaling.. J Biol Chem.

[27] Lapointe J, Li C, Higgins JP, van de Rijn M, Bair E (2004). Gene expression profiling identifies clinically relevant subtypes of prostate cancer.. Proc Natl Acad Sci U S A.

[28] Yeoh EJ, Ross ME, Shurtleff SA, Williams WK, Patel D (2002). Classification, subtype discovery, and prediction of outcome in pediatric acute lymphoblastic leukemia by gene expression profiling.. Cancer Cell.

[29] van de Vijver MJ, He YD, van 't Veer LJ, Dai H, Hart AA (2002). A gene-expression signature as a predictor of survival in breast cancer.. N Engl J Med.

[30] Beer DG, Kardia SL, Huang CC, Giordano TJ, Levin AM (2002). Gene-expression profiles predict survival of patients with lung adenocarcinoma.. Nat Med.

[31] Bhattacharjee A, Richards WG, Staunton J, Li C, Monti S (2001). Classification of human lung carcinomas by mRNA expression profiling reveals distinct adenocarcinoma subclasses.. Proc Natl Acad Sci U S A.

[32] Fisher RA (1925). Applications of “Student's” distribution.. Metron.

[33] Mani KM, Lefebvre C, Wang K, Lim WK, Basso K (2008). A systems biology approach to prediction of oncogenes and molecular perturbation targets in B-cell lymphomas.. Mol Syst Biol.

[34] Agresti A (1990). Categorical data analysis.

[35] Swets JA, Dawes R, Monahan J (2000). Psychological Science Can Improve Diagnostic Decisions.. Psychological Science in the Public Interest.

[36] Gambhir SS (2002). Molecular imaging of cancer with positron emission tomography.. Nat Rev Cancer.

[37] Gatenby RA, Gillies RJ (2004). Why do cancers have high aerobic glycolysis?. Nat Rev Cancer.

[38] Gatenby RA, Gillies RJ (2007). Glycolysis in cancer: a potential target for therapy.. Int J Biochem Cell Biol.

[39] Banka CL, Lund CV, Nguyen MT, Pakchoian AJ, Mueller BM (2006). Estrogen induces lung metastasis through a host compartment-specific response.. Cancer Res.

[40] Chen J, Yuan B (2006). Detecting functional modules in the yeast protein-protein interaction network.. Bioinformatics.

[41] Ideker T, Ozier O, Schwikowski B, Siegel AF (2002). Discovering regulatory and signalling circuits in molecular interaction networks.. Bioinformatics.

[42] Segal E, Wang H, Koller D (2003). Discovering molecular pathways from protein interaction and gene expression data.. Bioinformatics.

[43] Sharan R, Suthram S, Kelley RM, Kuhn T, McCuine S (2005). Conserved patterns of protein interaction in multiple species.. Proc Natl Acad Sci U S A.

