# Supplementary material for: Inferring Pathway Activity toward Precise Disease Classification
Source: PLoS Comput Biol. 2008 Nov 7;4(11):e1000217. doi: 10.1371/journal.pcbi.1000217 (PMC2563693; doi:10.1371/journal.pcbi.1000217)
Supplement: Table S1 — The seven data sets used in method evaluation (0.01 MB PDF) [file pcbi.1000217.s001.pdf]

Table S1. The seven data sets used in method evaluation.

Name	Publication	Number of samples in each class
NF-kB	Tian et al. 2005a [26]	Wildtype: 12 samples<br>Mutant: 12 samples
Prostate cancer	Lapointe et al. 2004 [27]	Normal: 41 samples<br>Cancer: 62 samples
Leukemia	Yeoh et al. 2002 [28]	TEL-AML1: 79 samples<br>HH: 64 samples
Breast_Netherland	van de Vijver et al. 2002 [29]	Metastatic: 78 samples<br>Non-metastatic: 217 samples
Breast_USA	Wang et al. 2005 [5]	Metastatic: 106 samples<br>Non-metastatic: 180 samples
Lung_Boston	Subramanian et al. 2005 [30]	Poor prognosis: 31 samples<br>Good prognosis: 31 samples
Lung_Michigan	Subramanian et al. 2005 [31]	Poor prognosis: 24 samples<br>Good prognosis: 62 samples
