# Supplementary material for: Inferring Pathway Activity toward Precise Disease Classification
Source: PLoS Comput Biol. 2008 Nov 7;4(11):e1000217. doi: 10.1371/journal.pcbi.1000217 (PMC2563693; doi:10.1371/journal.pcbi.1000217)
Supplement: Table S2 — Frequently selected pathway markers for breast cancer prognosis (0.01 MB PDF) [file pcbi.1000217.s002.pdf]

Table S2. Frequently selected pathway markers for breast cancer prognosis.

Pathway Name	Frequency	# genes *	CORGs
**From Netherlands to USA**			
Cyclin regulated genes	416/500	2/13	E2F1 *CCNE2*
IL7 pathway	200/500	3/16	BCL2 STAT5A IL7
Cell cycle	197/500	3/84	E2F1 *ESPL1* CCNB2
ActinY pathway	142/500	3/19	PIR PSMA7 ACTR3
GNF female genes	123/500	3/85	RPS4X RPS6 RPL6
**From USA to Netherlands**			
Cell Cycle	500/500	4/84	CCNE2 *ESPL1* MAD2L1 CDK2
Brentani cell cycle	282/500	4/86	CCNE2 MAD2L1 CDK2 MXD1
Cyclin regulated genes	280/500	3/13	*CCNE2* CDK2 CCNA2
KRAS up-regulated genes	202/500	3/84	TUFT1 P4HA2 COL4A1
Cell cycle checkpoint II genes	200/500	2/10	CCNE2 FANCG
Glutamine down-regulated genes	167/500	6/313	TCEB1 KPNA2 CYCS TMED9 UTP18 MORF4L2
MMP/Cytokine connection	148/500	5/15	DEAF1 TNFRSF1B CD44 IL1B TGFB2
Leucine down-regulated genes	136/500	6/180	TCEB1 KPNA2 CYCS TDG CCT6A CSE1L
IL22 pathway	124/500	3/13	SOCS3 STAT5A STAT3
Rapamycin down-regulated genes	111/500	4/229	STAU1 CYCS RAE1 MORF4L2

* The number of CORGs and member genes are specified.

** Pathways/Genes in italics are shared between datasets
