# Supplementary figures and images for: Inferring Pathway Activity toward Precise Disease Classification
Source: PLoS Comput Biol. 2008 Nov 7;4(11):e1000217. doi: 10.1371/journal.pcbi.1000217 (PMC2563693; doi:10.1371/journal.pcbi.1000217)

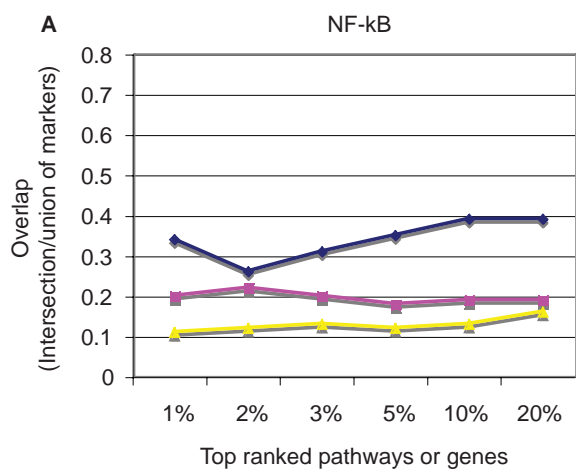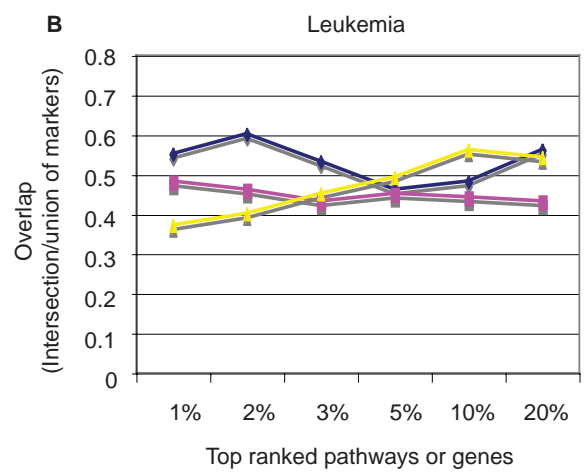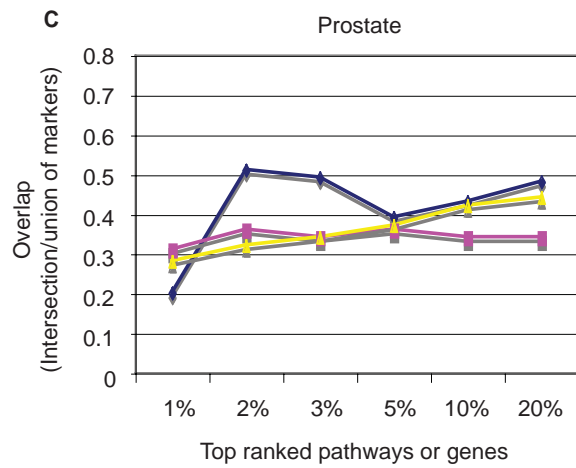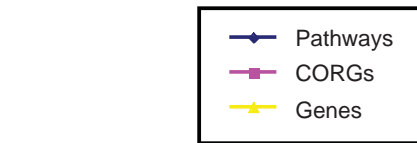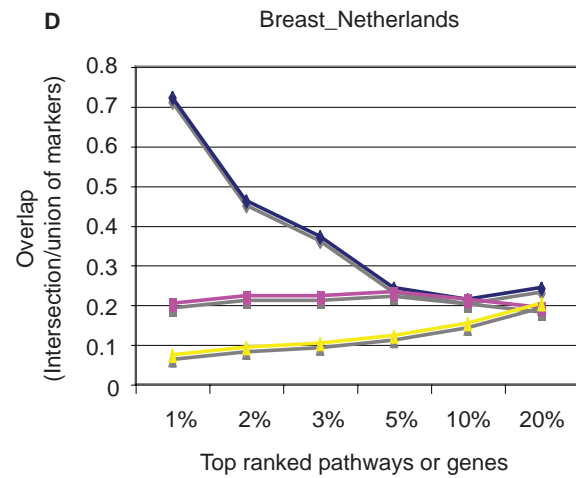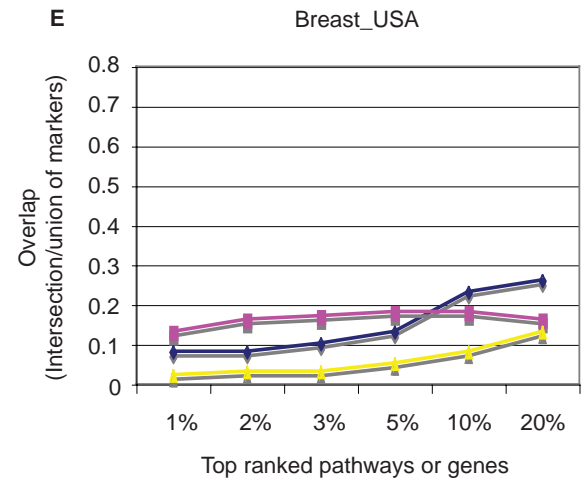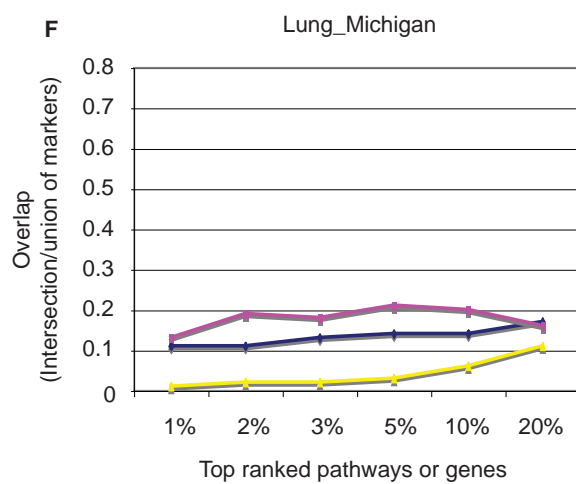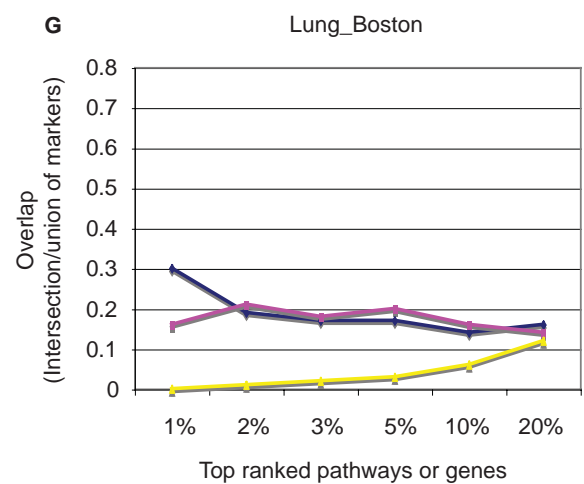

Supplement: Figure S1 — Marker reproducibility of pathway-based and gene-based selection in (A) NF-kB dataset, (B) Leukemia dataset, (C) Prostate dataset, (D) Netherlands dataset, (E) USA dataset, (F) Michigan dataset, and (G) Boston dataset Blue and yellow lines chart the magnitude of overlap among top n markers for pathways ranked by Tian et al. [16] and genes ranked by conventional t-test, respectively. Purple lines chart the magnitude of overlap among CORGs for the top n pathways. The performance of the 100 alternative splits is denoted by its mean. (0.03 MB PDF) [file pcbi.1000217.s003.pdf]

A

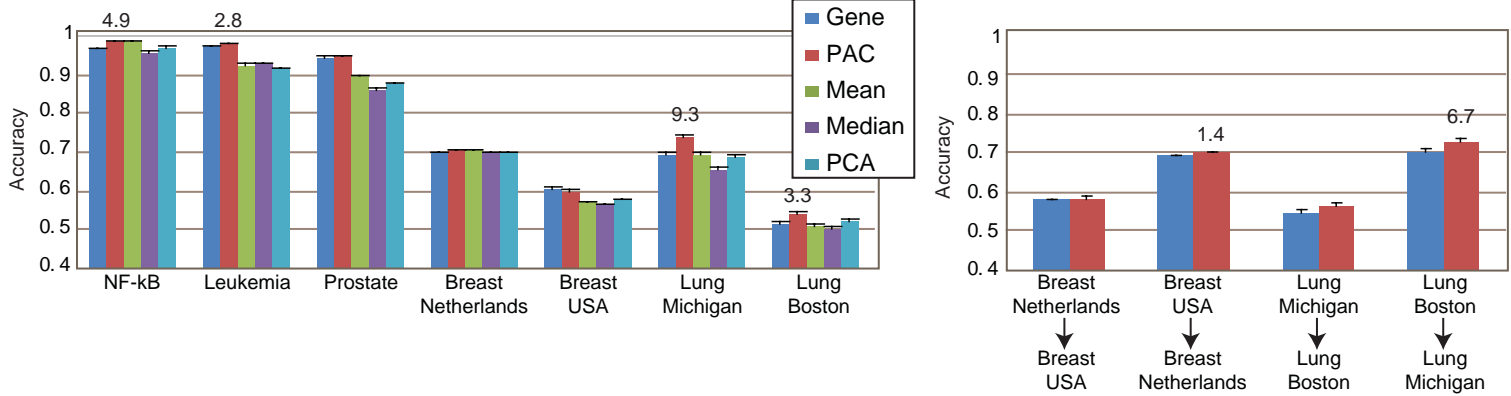

B

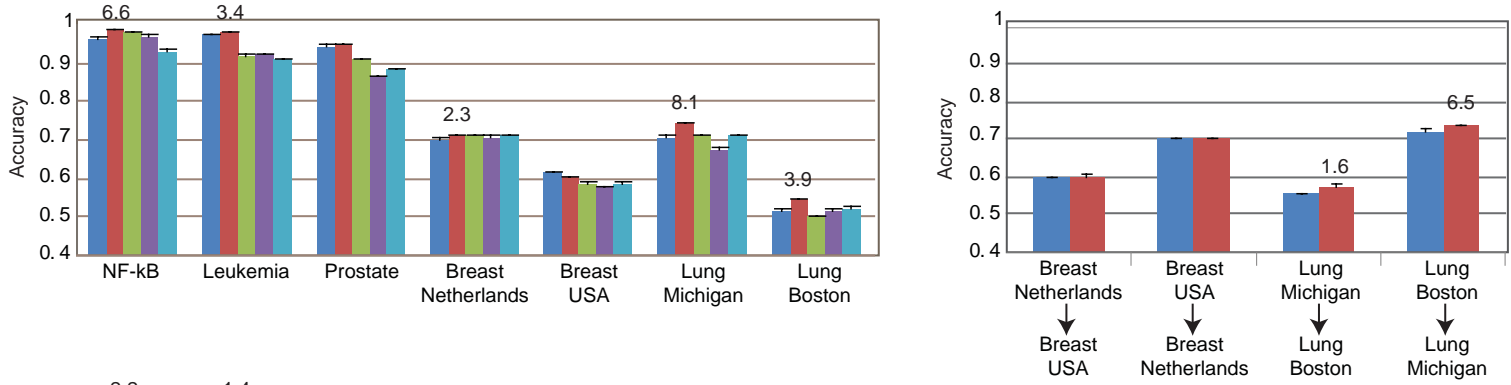

C

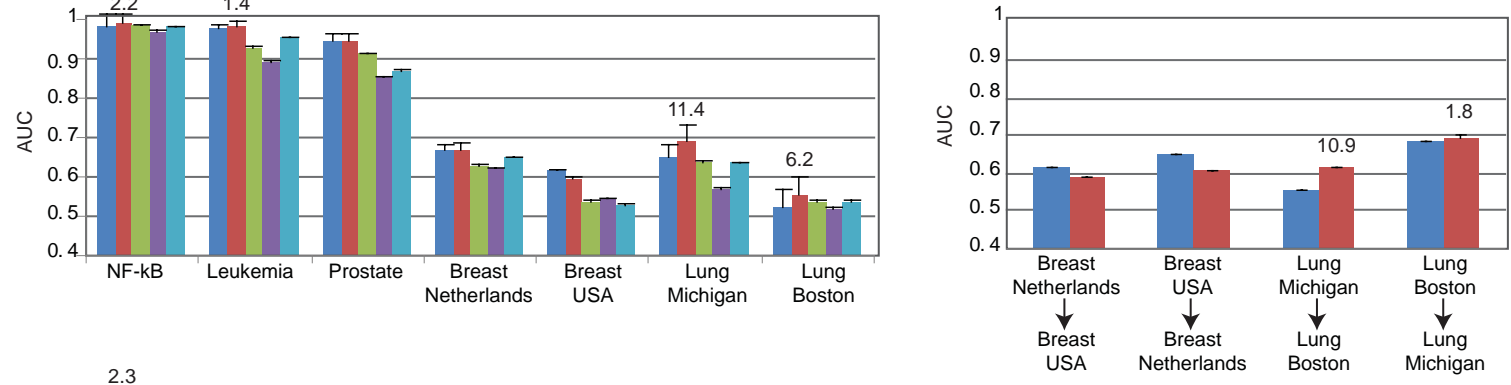

D

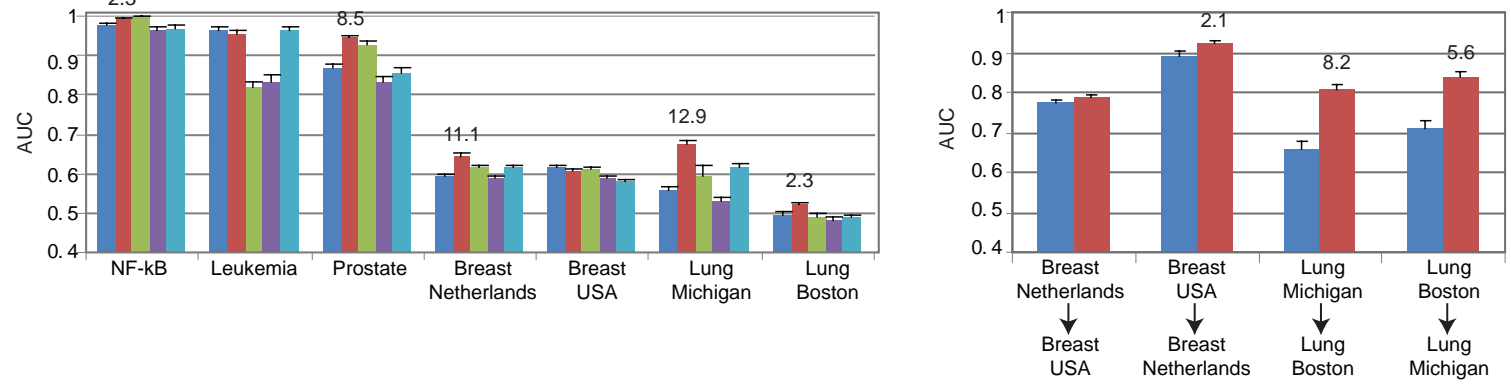

Supplement: Figure S2 — Classification accuracy within and across datasets using different classifiers, (A) k-nearest neighbor with k = 3, (B) k-nearest neighbor with k = 5, (C) naïve Bayes and (D) linear discriminative analysis Bar charts denote classification accuracy in (A) and (B) and Area Under ROC Curve (AUC) in (C) and (D). Classification performance is summarized as mean +/− ste of accuracies/AUCs over 100 runs of 5-fold cross-validation. Numbers above the red bars are -log (p-value) from the Wilcoxon signed-rank test on the 500 accuracies/AUCs of “PAC” against those of “Gene” (only the ones with p-value<0.05 are shown). (0.02 MB PDF) [file pcbi.1000217.s004.pdf]

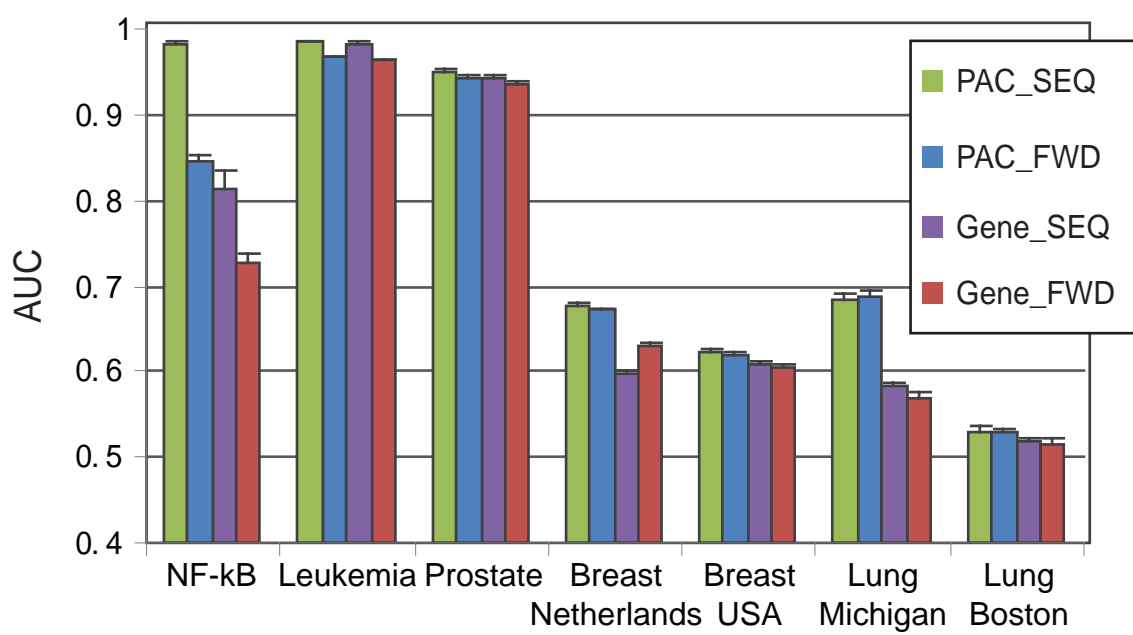

Supplement: Figure S3 — Classification performance using sequential selection (SEQ) or forward selection (FWD) (0.01 MB PDF) [file pcbi.1000217.s005.pdf]

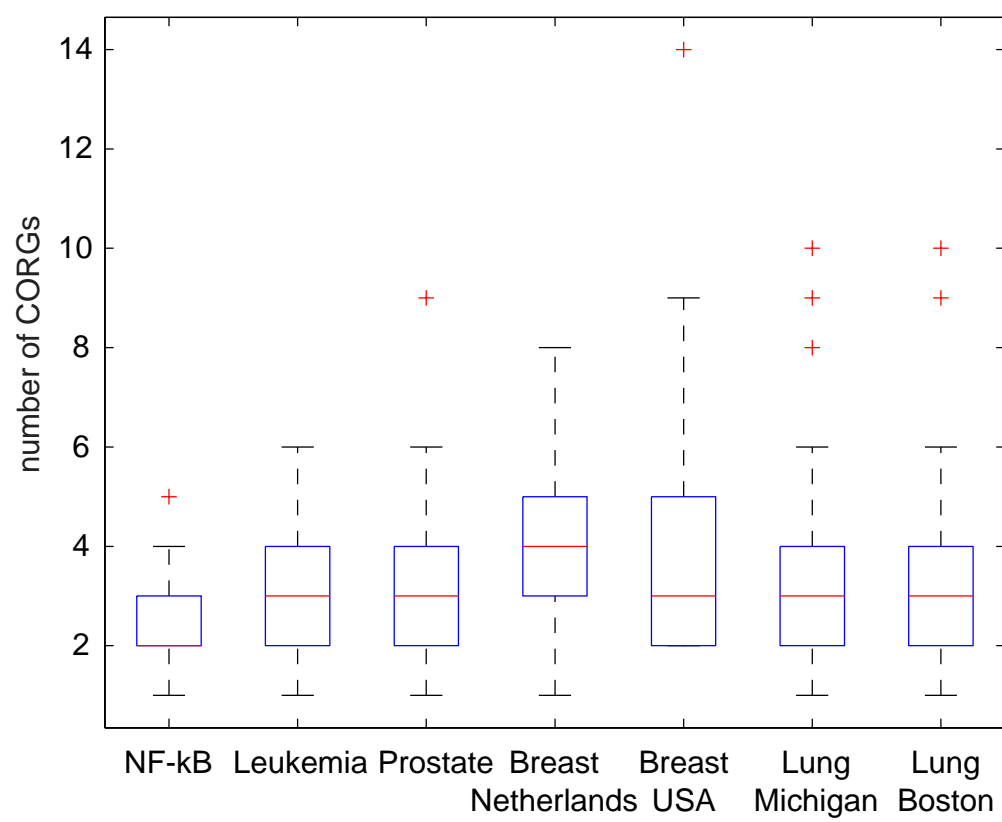

Supplement: Figure S5 — Distribution of numbers of CORGs in top 10% pathways (0.01 MB PDF) [file pcbi.1000217.s007.pdf]
