# Supplementary material for: Inferring Pathway Activity toward Precise Disease Classification
Source: PLoS Comput Biol. 2008 Nov 7;4(11):e1000217. doi: 10.1371/journal.pcbi.1000217 (PMC2563693; doi:10.1371/journal.pcbi.1000217)
Supplement: Figure S4 — Pathway activity of the top frequently used markers in the two breast cancer datasets Activities were inferred from CORGs identified from each dataset. Green/red blocks indicate pathways (rows) that are up-/down- regulated in patients (columns) of specific phenotype (above color bars: pink and green indicate metastasis and non-metastasis, respectively). Pathways are clustered based on the similarity of their activities across patients. (0.11 MB PDF) [file pcbi.1000217.s006.pdf]

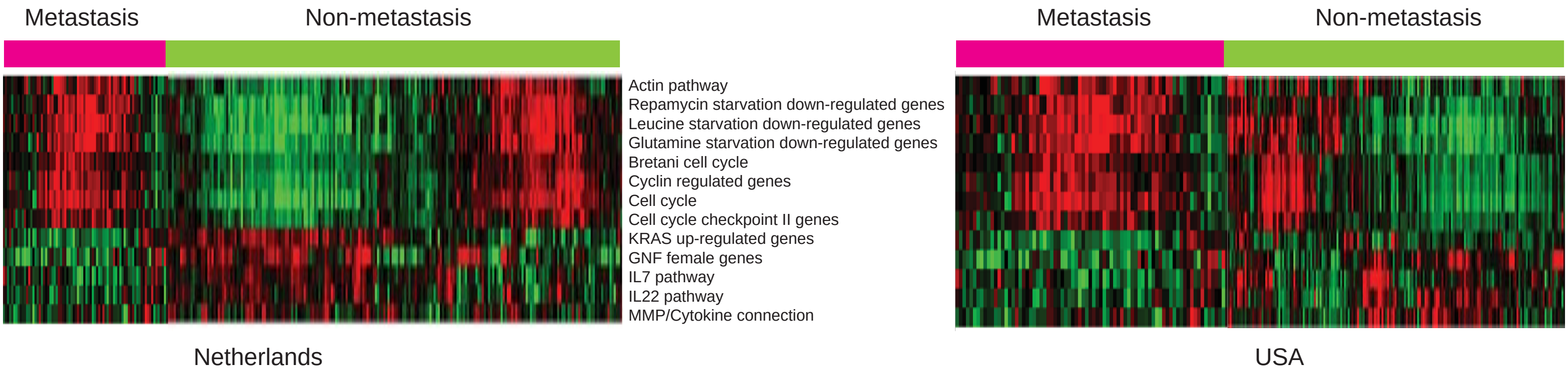
Metastasis
Non-metastasis
Actin pathway
Repamycin starvation down-regulated genes
Leucine starvation down-regulated genes
Glutamine starvation down-regulated genes
Bretani cell cycle
Cyclin regulated genes
Cell cycle
Cell cycle checkpoint II genes
KRAS up-regulated genes
GNF female genes
IL7 pathway
IL22 pathway
MMP/Cytokine connection
Netherlands
Metastasis
Non-metastasis
USA
